# Strategic sustainability practices in intercropping-based family farming systems: study on rural communities of Iran

**DOI:** 10.1038/s41598-023-45454-z

**Published:** 2023-10-24

**Authors:** Pouria Ataei, Afshin Mottaghi Dastenaei, Hamid Karimi, Nasim Izadi, Meysam Menatizadeh

**Affiliations:** 1 Department of Agricultural Extension and Education, Faculty of Agriculture, Tarbiat Modares University, Tehran, Iran; 2 Department of Political Geography, Faculty of Geographical Sciences, Kharazmi University, Tehran, Iran; 3 Department of Agricultural Extension and Education, Faculty of Agriculture, University of Zabol, Zabol, Iran; 4 Department of Agricultural Extension and Education, Faculty of Agriculture, Bu-Ali Sina University, Hamedan, Iran; 5 Department of Agricultural Extension and Education, School of Agriculture, Shiraz University, Shiraz, Iran

**Keywords:** Climate sciences, Environmental social sciences

## Abstract

This paper reports a realistic analysis of a region using Grounded Theory (GT) to provide a sustainable model for family farming systems based on the intercropping system in rural communities of Iran. Furthermore, the fuzzy analytic hierarchy process (FAHP) was applied to assign weights to the criteria and sub-criteria of intercropping and monocropping systems. According to the model, the main phenomenon was “sustainability in the family farming system based on intercropping”. In this model, the causal factors were found to include behavioral and attitudinal motivators. Micro- and macro-factors were identified as the interfering factors in family farming systems based on intercropping. Social factors, economic components, and environmental potentials were the contextual factors of this system. Finally, the consequences included the conceptual development and evolution of sustainability, socioeconomic transformation, and ecological-environmental transformation. The results of FAHP showed that the environmental criterion was ranked the first among all criteria underpinning the sustainability of the intercropping system.

## Introduction

In the last century, agricultural development negatively affected the earth's ecosystem due to technical growth and the use of chemical inputs. Especially, in developing countries, the surpass of population growth from food production and conventional intensive farming have led to food insecurity^1,2^ and environmental degradation^3–5^. In this situation, sustainable agriculture is not only worth pursuing but also inevitable^6^ because the relationship between agricultural development and the environment is a bilateral relationship, and neglecting the environment will inhibit the achievement of development goals^7^.

Sustainable development of agricultural systems is crucial for ensuring global food security^8^, natural resource conservation^9^, and social resilience^10^. Therefore, the coordination of food production, eco-environment, and farmers’ well-being is a core element in achieving sustainable development goals^11^. The goal of the sustainable development strategy is to create sustainable systems of systematic production that do not conflict with environmental, economic, and social interests and emphasize the conservation of resources^12^. In a sustainable agriculture system, farmers can increase crop supply in line with population growth and economic growth and with respect to environmental considerations^13^.

So far, different systems have been proposed for improving economic performance and environmental conservation, including organic farming, mixed crop-livestock farming, precision agriculture, and conservation agriculture^14^. Among the sustainable farming systems, the intercropping system is of particular importance. It has been a promising promotion in farming in developing countries^15–17^ and is an option for stakeholder farmers in risky situations^18^. Intercropping is considered one of the pillars of sustainability in crop systems. The use of natural principles of plant diversity at farms and good pest and weed management practices are more fruitful and sustainable in intercropping systems than in monoculture systems^19,20^. Intercropping as the association of different species at the same time and in the same space has been proposed to counteract the negative side effects of intensive monocropping^21^.

In other words, conventional farming through monocropping systems threatens the sustainability of agricultural production systems because it depends mostly on the use of chemical fertilizers and pesticides for higher crop yields and crop protection against pests and diseases with subsequently serious on-site and off-site environmental problems^22^. Conventional monocropping as a strategy for increasing crop yields has been associated with the degradation of the agroecosystems due to less biodiversity, water and soil pollution, high erosion rates, and so on^21^. The priority in post-modern agriculture is to ensure productivity and minimize negative environmental impacts^23^. So, sustainable agricultural production systems characterized by crop diversification, intercropping, and less use of chemical fertilizers and pesticides have been established as an important option for sustainable food production^22^. Intercropping systems aim to create ecological balance, increase quantitative and qualitative yields, reduce damage by pests, diseases, and weeds, and reduce farmers’ dependence on pesticides^24^. Intercropping has been suggested as a solution to increase production in sustainable agriculture and adaptation to climate change by increasing the number of species per unit area^25,26^. In this regard, scholars^27^ have suggested to farmers and government suitable spatial arrangements of an intercropping system that can be adopted to promote the development of sustainable agriculture. The broccoli-fava bean intercropping system could be an agroecologically viable alternative for sustainable agriculture^22^. Some scholars^22^ concluded that maize intercropping at a rate of 75% of the plant density with an improved Gachena variety enhanced the productivity and economic return of the system. Research^28^ determined that intercropping increased crop yields and reduced runoff and soil degradation. Intercropping also increases soil nitrogen and carbon and has a positive impact on the activity of soil enzymes^29^.

Intercropping systems have been widely applied worldwide due to their high economic benefits and land-use efficiency^27^, e.g., in Germany^30^, China^31^, and Brazil^32^. Several studies have shown that compared to monocropping, crop diversification and intercropping can effectively increase the resistance and resilience of agroecosystems to drought and disease incidences^33^, reduce the risk of crop loss^34^, reduce the use of external fertilizers, increase soil fertility^35^, optimize plantation structure^36^, improve land use efficiency^35^, and improve water use efficiency, nitrogen use efficiency, and field productivity^37,38^.

The literature shows that in Iran which is located in an arid-semiarid climatic zone, agriculture development is facing serious challenges in resources, the social sector, the economic sector, and physical resources management^39^. The environment in this country has suffered severe threats such as desertification, degradation of forests and pastures, land use change, low groundwater levels, and land drainage^40,41^. Besides adverse environmental conditions, the unsustainability of the family farming system in Iran, which has emerged as low economic productivity of this system in producing some crop^42^, its low adaptability to climate change^40^, and the instability to supply the food security of Iranian families, has made many researchers to suggest intercropping as an appropriate strategy for sustainable agriculture development in Iran because it can efficiently use inputs such as water and nutrient elements and prevent environmental degradation^43–45^.

Therefore, concerning the fundamental problem of instability in Iranian family farming systems, the main purpose of this study is to model sustainable development in these systems based on the intercropping system. In this regard, this study was conducted with a realistic analysis of the region and data collected through survey and Grounded Theory (GT) to provide a sustainable model for family farming systems based on the intercropping system. GT is a structured, yet flexible methodology. This methodology is appropriate when little is known about a phenomenon; the aim being to produce or construct an explanatory theory that uncovers a process inherent to the substantive area of inquiry. One of the defining characteristics of GT is that it aims to generate a theory that is grounded in the data^46,47^. GT is also defined as a set of integrated conceptual hypotheses systematically generated to produce an inductive theory about a substantive area^48^.

Furthermore, the fuzzy analytic hierarchy process (FAHP) was applied to assign weights to the criteria and sub-criteria of intercropping and monocropping systems (sustainability dimensions: environmental, economic, and social). The analysis is a multi-criteria technique that provides useful support in the choice among several alternatives with different objectives and criteria. The FAHP method has been used in determining the weights of the criteria by decision-makers and then ranking the methods determined by the conventional AHP method. The procedure of building a fuzzy AHP model follows establishing a comparison matrix, aggregating multiple judgments, measuring the consistency, and defuzzifying the fuzzy weights.

## Research method

This research is a developmental and applied study in terms of purpose and a qualitative study in terms of paradigm in which grounded theory was used as the method. Grounded theory is a qualitative method introduced by Glaser and Strauss^46^. This method is a kind of field research that examines and describes phenomena in their natural position. Another key feature of this method is the continuous comparison analysis in data collection and analysis^47^. The research population included intercropping stakeholders in Fars province, Iran. The dominant crops of the region are wheat, barley, and corn. In regions where intercropping is exercised, various types of cultivars are cultivated with wheat, barley, and corn. The research used first snowball and then theoretical sampling techniques. A total of 91 farmers participated in the first phase. The data collection was continued through unstructured interviews and group discussions until data saturation (theoretical saturation). Unstructured interviews were conducted with follow-up questions and continued until theoretical saturation was achieved^48,49^. Theoretical saturation refers to the point in data collection when no additional issues or insights emerge from data and all relevant conceptual categories have been identified, explored, and exhausted^47^. Before the start of each interview, the farmers were informed about the research goal, the confidentiality of the information, and the recording of the interviews. The interviews began with open questions. Then, the following questions were asked to access more enriched data based on the type of responses provided by the participants. The average interview time was approximately 35 min. All interviews were transcribed word by word in 24 h. Data were analyzed by coding through the three stages of open coding, axial coding, and selective coding^47^. It was also necessary to use the sweeping process during the coding process. Open coding is the analytic process by which concepts (codes) are attached to the observed data and phenomenon during qualitative data analysis. Axial coding is the second step of coding that follows open coding. In contrast to open coding where it breaks the data into discrete parts, axial coding begins to draw connections between codes. Axial coding organizes the codes it developed in open coding. Selective coding is the last step in grounded theory, where it connects all categories together around one core category. In doing so, it defines one unified theory around the research^47^. In the reciprocating step, the researchers should go back to the previous coding stages to revise them.

To ensure validity and reliability, the data were collected and analyzed through long-term contact with the participants and spending time with them. Also, multiple methods were used to collect information. The data were collected through interviews and field memos. The encoded interviews were also reviewed by the participants. As such, after encoding the interviews, some of them were returned to the participants to ensure that the codes corresponded with their experiences. To ensure the stability of the data analysis process, the codes were peer-reviewed. In this regard, the text of interviews, codes, and the collected classes were sent to the research committee and people familiar with qualitative research to collect their views on the accuracy of the analysis and the interpretation process. Also, when coding the interviews, a reference was made to the previously encoded interviews to revise the codes. To increase the transferability of the findings, with the accurate and targeted description of the research process, it was also possible to follow the research process for others.

In the second phase, FAHP was used to assign weights to the criteria and sub-criteria of sustainability. It was, indeed, applied to pairwise comparison and ranking of the criteria and sub-criteria of intercropping and monocropping systems. In this phase, 205 farmers (62 farmers in intercropping systems and 143 farmers in monocropping systems) were selected by stratified random sampling with the proportional allocation as the research sample (the sample size was determined using Krejcie and Morgan’s (1970) table). In other words, criteria and sub-criteria of sustainability were ranked by farmers in intercropping and monocropping systems, separately. FAHP facilitates the process of competitive advantage selection and is used to perform a series of sensitivity analyses. It can adequately handle the inherent uncertainty and imprecision of the human decision-making process and can provide the flexibility and robustness needed for the decision-maker to understand the decision priorities in intercropping and monocropping systems. The details of the FAHP implementation include:

1. Weighting the criteria and sub-criteria: After the main criteria, F_i_ (F_1_, F_2_, …, F_n_), and sub-criteria, C_j_ (C_1_, C_2_, …, C_n_), are specified, their weights (WC_ij_ and WF_i_) are determined.
2. Determining the fuzzy relative importance of each individual alternative versus each individual sub-criteria: The alternatives, A_1_ (A_1_, A_2_, …, A_k_), are assessed with respect to each criterion, C_j_ (C_1_, C_2_, …, C_n_). Finally, their fuzzy relative importance, _ij_AC^1^, is determined.
3. Since the sub-criteria may differ in value (positive or negative), they need to be made dimensionless before their application. In other words, the positive sub-criteria with the highest value should be ranked the highest and the negative sub-criteria with the lowest value should be ranked the lowest. To transform into fuzzy dimensionless sub-criteria, their relative weight (*AC*_*ij*_ = *l*_*ij*_*,* *m*_*ij*_*,* *u*_*ij*_) with a positive value is applied.

In a comparison matrix, which was presented by Saaty and Vargas^50^, the comparisons are made on a 1–9 scale. If the comparison matrix is inconsistent, we will have the following relation:

$${r}^{\mathit{rii}}-0.5rii+rii-1\frac{1}{\mathit{rii}}-1\left(\frac{1}{rii}-1\right)*\left(\frac{1}{rii}-1\right)$$

Theoretically talking, the membership scale is derived from Saaty’s scale by the following equation. After Saaty’s scale was transformed into a fuzzy number by $r_{ij} = \frac{{a_{ij} }}{{a_{ij} + 1}}$, the membership scales were calculated for each individual criteria and sub-criteria. Then, the following equations were used to calculate and prioritize the weights.

$$ \begin{gathered} W = (w_{1} ,w_{2} ,...,w_{n} ), \hfill \\ w_{i} = \frac{{b_{i} }}{{\sum\limits_{i = 1}^{n} {b_{i} } }}. \hfill \\ where,b_{i} = \frac{1}{{\left[ {\sum\limits_{j = 1}^{n} {\frac{1}{{r_{ij} }}} } \right] - n}} \hfill \\ \end{gathered} $$

Also, the consistency ratio (CR) of the comparison matrix was tested by the following equation.

$$\mathrm{CR}=\frac{CI}{RI}<0.1 CI=\sum_{i=1}^{n}\frac{\left(AW\right)1}{nw1}$$

## Research questions

In general, the research aimed to answer the following questions:

- What are the contextual factors for the sustainable development of intercropping-based family farming systems?
- What causal factors influence the sustainability of intercropping-based family farming systems?
- What are the intervening factors for the sustainable development of intercropping-based family farming systems?
- What strategies are there for the sustainable development of intercropping-based family farming systems?
- What will the consequences of developing intercropping-based family farming systems be?
- How are the sustainability dimensions ranked from the perspective of farmers based on intercropping and monocropping?

## Results

To derive a theory from the real characteristics of the family farming system based on intercropping, attempts were made to understand the internal structure of the participants' values, attitudes, and experiences. The core of the discussion (sustainability in family farming systems based on intercropping) was discovered during the interviews because all the participants explained the main theme, i.e., sustainability, in different dimensions of economic, social, and environmental. The collected data were analyzed by open, axial, and selective coding. In the first stage of open coding, the original sentences, called concepts, were first extracted from the transcripts of the interviews, and each was assigned a code with the letter W. In the next step, similar concepts were classified into different categories. In the next step, axial coding was done according to which the extracted categories were categorized into conditional, equilibrium, and consequential categories^51^.

In this model, the focus of the participants’ attention was on the main phenomenon; that is, the sustainability of this type of farming system. In other words, they argued how intercropping would lead to sustainability in family farming systems. Participants explained this phenomenon through its causal factors and stated what factors influenced the process of its formation, what the nature of the process was, and which intervening factors could affect it. Finally, the relationship between the main theme and the subthemes, the sustainability strategies, and their consequences in this system were identified. From the interviews, a total of 416 initial conceptual propositions were derived in the open coding stage and 52 theme-specific propositions in the axial coding step.

**Figure 1:**
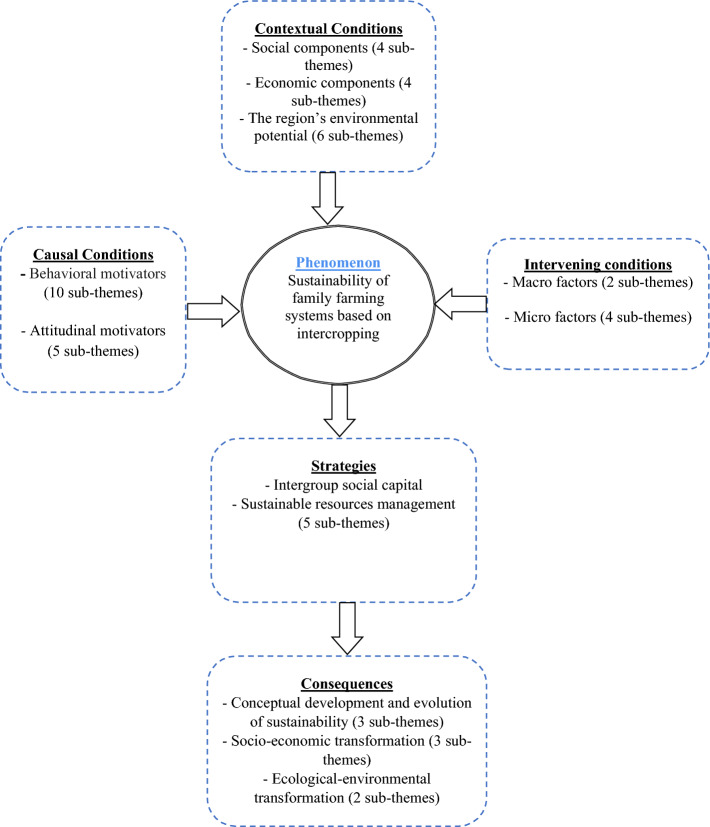
The paradigm model of sustainability of family farming systems based on intercropping.

The conditional themes that are, in fact, the inputs to the model included behavioral and attitudinal motivators, macro- and micro-factors, social constructors, economic components, and environmental potentials. Interactive process factors (strategies) included intergroup social capital and sustainable resource management. Eventually, the consequences included the conceptual development and evolution of sustainability, socio-economic transformation, and ecological-environmental transformation. After the axial coding step, selective coding was used to combine and integrate the categories from the previous steps to design the conceptual framework of the research (Fig. 1).

### Causal factors

The causal factors are those that directly contribute to sustainability in intercropping-based farming systems. They are composed of two categories: behavioral motivators and attitudinal motivators.

#### Behavioral motivators

Behavioral motivators are factors that lead stakeholders to specific crop behaviors or activities, such as intercropping. For example, as long as the stakeholders believe that this type of crop activity has no added value to them as economic, social, or biological sustainability, they will not be motivated to use it. In other words, this cropping system must have the necessary effectiveness for farmers. Creating full-time employment for the entire household, making more money than the monoculture system, diversifying the household basket, providing livestock products using product remnants, needing no product storage and ensuring the quick sale of products, replacing degraded products with alternative products, having simple and low-cost access to energy resources, supplying water from wells, and supplying power are the most important sub-themes of behavioral motivations in intercropping for farmers. For example, a participant (Farmer No. 9) said, ‘*The use of crop residues as fodder provided an incentive to change our cropping method into intercropping*’ (W_13_). Another participant (Farmer No. 51) said, ‘*When you have cultivated several crops and you’ll need less space for the storage, you’ll have the motivation to change your cultivation method*’ (W_43_).

#### Attitudinal motivators

Attitudinal motivators refer to farmers' mental expectations for the sustainable farming of the family farming system. In other words, this cultivation system should guarantee sustainability aspects for stakeholders logically. For example, cash sales of the crop by cultivating different crops (cucumber, beans, watermelon, corn, and sunflower), stakeholders' protection from economic risks (replacement of a product for a degraded product), improving the quality of life, reducing poverty and socioeconomic vulnerability, and creating cultural development opportunities for households (continuing education of children in higher education) provide the necessary rationale in the mindset of the farmers for an intercropping system. For instance, as a farmer (Farmer No. 79) asserted, ‘*When I apply intercropping in my farm, I feel that I’m posed to less economic risk. This changes my view on changing my farming method to intercropping*’ (W_71_). Also, Farmer No. 52 added, ‘*Since I realized that I can grow crops that I can sell in cash, my view on the traditional farming system was changed*’ (W_49_).

### Contextual factors

Such a phenomenon as the sustainability of intercropping-based farming systems can be achieved in specific environmental conditions provided that the appropriate conditions are available, not in a vacuum. The contextual conditions consist of three categories: (1) structural social components; (2) economic components; and (3) environmental potentials of the region.

#### Structural social components

Structural social components are the most important constructs in the sustainability of intercropping-based farming systems. Stakeholders’ satisfaction with their jobs, the use of synergistic force of participation in crop processes, and the exchange of information and technical knowledge among other farmers are the social characteristics of farmers who are bound by the sustainability of the system. For example, an intercropping farmer (Farmer No. 66) noted, ‘*The support of experts and pioneering farmers made me think about changing my cultivation method*’ (W_203_).

#### Economic components

The sustainability of the inter-cropping system is also dependent on economic indicators and material benefits. Increasing crop yields per unit area, increasing household incomes, reducing production costs, and reducing production waste have been the economic sub-themes in the sustainability of the intercropping system. Farmer No. 1, for example, said, ‘*In the first year of intercropping, I produced more crops and this made me keep on intercropping in the next year*’ (W_12_).

#### Environmental Potentials of the Region

Undoubtedly, the sustainability of an intercropping system in the region requires fitting environmental characteristics and potentials with the characteristics and requirements of this farming system. Subcategories such as the suitability of land drainage, the health of the mechanical texture of the soil due to the lack of tillage operations, the suitability of soil texture in terms of the combination of soil elements due to crop rotation, the suitability of the physical texture of the soil due to the non-burning of the crop residues, the suitability of the chemical texture of the soil due to the use of animal fertilizers, and high soil production capacity due to limited use of chemicals (herbicides, fungicides, and chemical fertilizers) have been among the most important environmental sub-themes in the sustainability of intercropping systems. For example, Farmer No. 62 pointed out, ‘*Experts checked my land and said that its soil was suitable for intercropping and I could implement it*’ (W_379_).

### Strategies

Although the causal and contextual factors provide the necessary conditions for the sustainability of intercropping farming systems, they are not considered sufficient. In fact, in the absence of appropriate strategies, the causal and contextual factors may not be able to produce the phenomenon. Strategies cover two themes: (1) intergroup social capital and (2) sustainable resource management.

#### Intergroup social capital

The continuity and sustainability of a particular cultivation method, first of all, require the group normative, solidarity, and responsibility spirits. Family cohesion in using the physical power and skills of all household members in the process of cultivation, interaction, and support of household members in the timely harvest of the crop to prevent the loss of crop quality, the existence of strong links between household members and their commitment, and accountability to each other are some requirements for creating inter-group social capital which ensures the sustainable production process in intercropping-based family farming systems. For instance, a farmer (Farmer No. 39) stated, ‘*Intercropping caused me to ask my family members to give me a hand both in the planning and the harvesting steps*’ (W_119_).

#### Sustainable Resource Management

Comprehensive and rational management strategies in human resources management, energy resources, ecological and climate resources, and market demand management are among other factors contributing to the continuity and sustainability of productivity in intercropping-based farming systems. Cropping diverse and seasonal products for the purpose of employing full-time members of the household, optimal use of energy resources (water, electric motor power, crop residues as a fuel, and energy generation source), flexibility in changing the cropping pattern in terms of creating ecological balance, the proportion of cultivation with rainfall pattern and climatic conditions of the region, and changing crop type in accordance with market demand are some of the most important management sub-themes in the sustainability of intercropping-based family farming systems. For example, Farmer No. 59 stated, ‘*In the intercropping system, the crop is selected in accordance with the market and rainfall to, as the experts said, allow for sound management of the farm*’ (W_251_).

### Intervening factors

Intervening factors constitute effective and interactive actions in the sustainability of intercropping-based family farming systems. Intervening factors consist of two categories: (1) macro-factors and (2) micro-factors.

#### Macro-factors

Although the sustainability of intercropping-based family farming systems is influenced by causal factors, contextual factors, and specific strategies, the role of human agency and actors should not be ignored. They can facilitate the processes with their interventions. In fact, actors act as mediators of change. This theme at the macro level includes the sub-themes, such as facilitator services and extenders of the Agricultural Jihad Organization (counseling services, educational services, and financial services such as low-interest loans and crop insurance). A farmer (Farmer No. 15), for example, said, ‘*If experts did not visit the farm and guide us, we would stick to our traditional farming method*’ (W_169_).

#### Micro-factors

The sustainability of intercropping-based family farming systems requires the presence of factors at the micro level or at the level of system stakeholders. Traditionalism and the desire to preserve the traditions of fathers in agricultural management, the religious beliefs of farmers, and the indigenous knowledge of local people are among the themes raised by the respondents as effective regional catalysts for adherence to these systems. For instance, Farmer No. 16 pointed out, ‘*If we were to do farming as per our ancestors’ traditions, we would have to abandon this profession by the next years because water and soil are destroying and other agricultural inputs, such as fertilizers and pesticides, are very expensive*’ (W_191_).

### Consequences and results

Grounded Theory follows an “if, then, then” pattern in explaining the processes that have an impact on the sustainability of intercropping-based family farming systems. That is, if there are appropriate actions for environmental conditions and contextual conditions, the causal factors of the occurrence of the phenomenon (sustainability) are provided and we will then see the consequences of the occurrence of the phenomenon. The consequences include three themes: (1) conceptual development and evolution of sustainability, (2) socio-economic transformation, and (3) ecological-environmental transformation of sustainable intercropping-based farming systems.

#### Conceptual development and evolution of sustainability in family farming systems

The reconstruction of the concept of sustainability and the correct understanding of the nature and reasons for sustainability in intercropping-based family farming systems have been emphasized because sustainability is a holistic concept and is not merely limited to the non-use of chemicals, but, in a farming system as a system with all its constituent elements, it includes human, social, economic and environmental aspects. Such a view in the farmers of the farming system is the foundation of sustainable development that guarantees the endurance and sustainability of the aforementioned cropping system. For example, an interviewee (Farmer No. 64) noted, ‘*So far, I’ve thought that sustainable farming only means less chemical fertilizer and pesticide use, but I have realized that sustainable income is also a component of sustainable agriculture*’ (W_301_).

#### Socioeconomic transformation

Today, the role of sustainable crop systems in the socio-economic development of stakeholders is not overlooked given the scarcity of available welfare facilities, the trend of youth migration, the escalation of unemployment, and the occurrence of socioeconomic abnormalities. The dissemination of indigenous experiences and knowledge of farmers about the development of intercropping in order to enjoy the benefits of this system in the region and the emphasis of the stakeholders and the cooperation of government agencies and authorities on the formation of a network of farmers in the form of cooperatives and private associations to provide services to the stakeholders from crop production to crop sale stages have been the other important consequences in this research. These consequences will lead to significant socioeconomic transformations, including the creation of employment for households and, therefore, the prevention of immigration and the sustainable livelihoods of the family. Indeed, Farmer No. 90 stated, ‘*The shift to intercropping activated our rural cooperation and all farmers gathered around it*’ (W_381_).

#### Maintaining ecological balance

The overuse of the land, the excessive use of pesticides and fertilizers, and the inappropriate methods of exploiting soil and water resources to gain higher yields will pose major challenges in the long run although they may be plausible in the short run and may lead to an acceptable income. Some of these challenges include soil alteration, water and soil contamination, biodiversity loss, the prevalence of pests, diseases, and weeds, soil erosion, and production decline. Undoubtedly, considering the decrease in incomes from traditional agricultural activities and short-term monoculture systems and the lower productivity of production factors, the economic aspect of agricultural production will be challenged in the future. Therefore, given its role in crop diversification and its specific features, the development of an intercropping system will have numerous consequences such as less prevalence of pests, diseases, and weeds, farmers' lower dependence on pesticides, optimal use of natural resources, stabilization of soil elements due to the use of legumes in cultivation diversity, balance in soil elements, biomass increase, soil surface protection due to vegetation, and soil protection against wind and chilling in autumn crops, which would contribute to maintaining ecological equilibrium as another significant result emphasized by Grounded Theory in the present study. For example, Farmer No. 22 said, ‘*After several years of using intercropping, the soil at my farm has become more fertile, which is delightful*’ (W_81_).

### Ranking the criteria and sub-criteria of intercropping and monocropping systems

In the second phase of this study, the consequences (environmental, social, and economic) of the intercropping-based family farming systems were ranked as criteria and sub-criteria of sustainability dimensions. The criteria and sub-criteria of sustainability dimensions are illustrated in Table 1. The findings indicated that the criteria of environmental sustainability included eight sub-criteria: fertilizers, pesticides, efficient irrigation systems, crop diversity, water balance, conservation tillage, electrical conductivity, and crop rotation. Economic sustainability encompassed five sub-criteria of economic risk, the dependence of farmers on state aid, the level of employment, the gross value of production, and crop yield. Furthermore, four sub-criteria were derived for social sustainability. These sub-criteria include literacy, population density, education, and courses for sustainability promotion. Criteria and sub-criteria of sustainability were ranked by farmers in intercropping and monocropping systems, separately.

**Table 1:** Criteria and sub-criteria of sustainability dimensions.

Criteria	Sub-criteria	Definition
Environmental	Z1 Fertilizers	Consumption per unit area (kg/ ha)
	Z2. Pesticides	Consumption per unit area (liters per hectare)
	Z3. Efficient irrigation systems	The total area of land under modern irrigation
	Z4. Crop diversity	Formula H- degree of diversity of crop plants
	Z5.Water balance	Overuse of groundwater
	Z6. Conservation tillage	Low-tillage and no-tillage farms to total land area
	Z7. Electrical conductivity (EC)	Percent of total arable land acreage of saline lands
	Z8. Observe crop rotation	The principles of crop rotation
Economic	E1.Economic risk	Lands covered by insurance to total land
	E2. The dependence of farmers on state aid	The dependence of farmers on state aid
	E3. The level of employment	The proportion of the population employed in agriculture
	E4. The gross value of production	The average gross production value per hectare of arable land
	E5. Crop yield	Yield per hectare (kg)
Social	S1. The literacy	The ratio of literate farmers to the farmers in each region
	S2. Population density	The proportion of the population to the total area
	S3. Education	The proportion of extension agents to the population
	S4. Courses for sustainability promotion	The number of sustainability promotion courses

Table 2 shows the weighted value of criteria and sub-criteria in intercropping systems. The results revealed that the environmental criterion was ranked the first among all criteria underpinning the sustainability of the intercropping system. It was given a weighted importance of 0.445. The second and third ranks were for the economic criterion with a weighted importance of 0.387 and the social criterion with a weighted importance of 0.369, respectively.

**Table 2:** Weighted values of criteria and sub-criteria in intercropping systems.

Criteria and sub-criteria	Weighted value of criteria	Sub-criteria	Relative weight of sub-criteria	Final weight of sub-criteria
Environmental	0.445	Z1	0.142	0.069
		Z2	0.141	0.069
		Z3	0.152	0.068
		Z4	0.117	0.052
		Z5	0.127	0.057
		Z6	0.115	0.048
		Z7	0.068	0.038
		Z8	0.132	0.067
Economic	0.387	E1	0.138	0.055
		E2	0.177	0.065
		E3	0.198	0.077
		E4	0.198	0.078
		E5	0.297	0.118
Social	0.369	S1	0.227	0.039
		S2	0.112	0.019
		S3	0.312	0.054
		S4	0.341	0.068

Table 3 shows the weighted value of criteria and sub-criteria in monocropping systems. The results illustrated that the economic criterion was ranked the first among all criteria underpinning the sustainability of monocropping systems. It was given a weighted importance of 0.487. The second and third ranks were for the environmental criterion with a weighted importance of 0.305 and the social criterion with a weighted importance of 0.269, respectively.

**Table 3:** Weighted values of criteria and sub-criteria in monocropping systems.

Criteria and sub-criteria	Weighted value of criteria	Sub-criteria	Relative weight of sub-criteria	Final weight of sub-criteria
Environmental	0.305	Z1	0.124	0.068
		Z2	0.125	0.068
		Z3	0.135	0.065
		Z4	0.112	0.048
		Z5	0.123	0.055
		Z6	0.112	0.047
		Z7	0.064	0.029
		Z8	0.135	0.053
Economic	0.487	E1	0.149	0.056
		E2	0.168	0.068
		E3	0.192	0.073
		E4	0.191	0.077
		E5	0.297	0.116
Social	0.269	S1	0.228	0.039
		S2	0.109	0.017
		S3	0.293	0.055
		S4	0.332	0.064

To determine the final importance score of each criterion, the weighted importance of the sub-criteria was multiplied by that of the criteria. It should be noted that all items had Inconsistency Ratio (IR) < 0.01. In other words, IR is a measure of whether there is consistency between the pairwise comparisons in the questionnaires. It can be used to measure the consistency of the responses provided by participants for the assessments and pairwise comparisons. The results indicated that the intercropping system was ranked first versus the monocropping system (Table 4). It can be argued that the intercropping system was in line with sustainability. It should be mentioned that closeness (CL_i_) is the relative proximity index and score indices (Si + /−) is defined as distance from the positive/negative ideal option.

**Table 4:** Agricultural sustainability ranking.

Farming system	***Si*** +	***S***_***i***_-	***CL***_***i***_^∗^	Rank
Monocropping	0.054	0.093	0.620	2
Intercropping	0.068	0.108	0.627	1

## Discussion

The findings indicate that the causal factors are composed of two categories: behavioral motivators and attitudinal motivators. Some scholars^52–54^ have considered the role of motivations in accepting various measures of sustainable farming by farmers. It is important to recognize the incentives for farmers to choose crop variety^55^. Researchers have considered the formation of attitudes as affecting different behaviors. It has been argued that people's attitudes toward sustainability will be the basis for sustainability behaviors^56–59^.

The contextual conditions were found in three criteria: structural social components, economic components, and environmental potentials of the region. Researchers have also considered different social factors, such as participation among farmers^60,61^, social capacity building^62^, social support^63^, and knowledge sharing^64^ as effective in crop activities. Researchers^65–67^ believe that economic benefits have always been the main factors in selecting and adopting sustainability measures by farmers. Some scholars have also mentioned that the environmental conditions include, for example, soil texture^68^, crop rotation^69,70^, maintaining crop residue on the soil surface^71–73^, and using green manure^72,74^.

The findings lead us to understand that strategies cover two criteria: intergroup social capital and sustainable resource management. Researchers^61,75–77^ have also considered social capital and its elements such as accountability and cohesion among stakeholders. Furthermore, issues associated with resource exhaustion and climate change continue to worsen, and sustainable resource management is considered a method of resolving these problems^78^. In addition, a suitable approach should be developed for a sustainable resource management model that considers the interrelationships and provides a guideline for performance improvement^79^.

The results indicate that intervening factors consist of two categories: macro-factors and micro-factors. Researchers have also emphasized the extension of the role of promoting and providing farmers' training needs^80,81^, understanding conflicts among them^81–83^, and knowledge of climate change^84^. Some scholars^85–88^ have concluded that there was an important role for religiousness, spirituality**,** and indigenous and local knowledge in making decisions about the use of sustainable practices and their management.

The consequences and results also include three criteria of conceptual development and evolution of sustainability, socio-economic transformation, and ecological-environmental transformation of the intercropping-based sustainable farming system. Researchers^89–93^ have stated that there must be a common understanding of the concept of sustainability between farmers, policymakers, and researchers. Also, most scholars^94–98^ have argued that many of the environmental challenges could be solved by maintaining ecological-social equilibrium. Others also believe that land use efficiency^99^, tourism development^100^, human capital^101^, household income growth^102^, and interaction of internal and external systems^93^ in rural area leads to environmental sustainability.

## Conclusion

Intercropping is a type of sustainable farming practice that entails two or more crops growing in the same field side by side in the same or overlapping growing season. The economic and environmental values of intercropping systems in dually promoting food security and environmental health can serve as a basis for policy consideration as governments and stakeholders explore more sustainable farming options. The results indicated that three conditions (causal, intervening, and contextual conditions) can be effective in the sustainability of intercropping-based family farming systems. Accordingly, two strategies (intergroup social capital and sustainable resources management) should be considered for developing intercropping among family farming systems. The sustainability of the intercropping-based family farming system can be ensured by developing intra-group social capital at the farmer’s family level via creating a sense of belonging in the individual members to the family farming system, reinforcing trust and solidarity at the family level, improving members’ commitment to goals and practices of the intercropping system, responding to other members, observing ethics, and being responsible. Indeed, reinforcing intra-group social capital increases the participation of the farming system’s members in the sound and timely implementation of agronomic practices, which would entail the sustainable enhancement of the system's efficiency. As well, the capability of optimal and sustainable management of physical, financial, and human resources can be effective in increasing the productivity of the intercropping-based family farming system. Indeed, the limitations of resources and energy, e.g., water, land, and fuel, have necessitated scientific management. The enhancement of the knowledge and skills of the family farming system’s members about the sustainable management of available resources needs continuous learning from up-to-date information sources. Other scholars^103–107^ also emphasized on continuous and deep learning. These strategies will have many consequences, such as conceptual development and evolution of sustainability, socio-economic transformation, and ecological-environmental transformation. Also, the results showed that the environmental criterion was ranked first among other criteria. It means that the farmers perceived sub-criteria of environmental sustainability and the sub-criteria were the main components for choosing best practices and farming systems.

According to the results, it can be recommended that technical points (e.g. introducing drought-adaptive crops and training row intercropping to improve the productivity of fertilization, pesticide use, and harvest machinery) must be based on the farmers’ experiences and indigenous knowledge. The technical points can improve productivity and develop intercropping systems. Furthermore, farmers are recommended to get acquainted with the environmental, economic, and social advantages of intercropping development, which would extend the use of sustainability principles at their farms.

## Data Availability

The datasets generated during and/or analyzed during the current study are available from the corresponding author on reasonable request.
